# Smart Wearables for Cardiac Autonomic Monitoring in Isolated, Confined and Extreme Environments: A Perspective from Field Research in Antarctica

**DOI:** 10.3390/s21041303

**Published:** 2021-02-11

**Authors:** Michele M. Moraes, Thiago T. Mendes, Rosa M. E. Arantes

**Affiliations:** 1 Department of Pathology, Institute of Biological Sciences, Universidade Federal de Minas Gerais, Belo Horizonte, Minas Gerais 31270-901, Brazil; michelemmoraes@ufmg.br; 2 Center for Natural and Human Sciences, Health and Technology, Universidade Federal do Maranhão, Pinheiro, Maranhão 65200-000, Brazil; thiagotemendes@gmail.com

**Keywords:** cardiovascular, cold, isolation, polar, portable devices

## Abstract

Antarctica is a space-analog ICE (isolated, cold, and extreme) environment. Cardiovascular and heart autonomic adjustments are key-adaptive physiological responses to Antarctica, both in summer camps and in research stations winter-over. Research fieldwork in ICE environments imposes limitations such as energy restriction, the need for portable and easy-to-handle resources, and resistance of materials to cold and snow/water. Herein, we present the methods we use for cardiac monitoring in the Antarctic field, the limitations of the equipment currently available, and the specific demands for smart wearables to physiological and health tracking in ICE environments, including the increased remote monitoring demand due to COVID-19 restrictions.

## 1. Introduction

Moving to or staying in isolation, confinement, and extreme conditions (ICE) defy the human body [1,2,3]. Dynamic behavior of cardiac autonomic control is a key-adaptive physiological response to ICE, from Antarctica [4,5,6], passing by flight maneuvers [7], to a space station [8,9]. Antarctic ICE-related aspects vary in intensity and relevance depending on the environment (i.e., the prolonged ship travels, staying at camps, research stations, and refuges) [2,10,11,12], more often and intensely experienced in camps. During camping, the distress posed by the extreme light regimen (i.e., 24 h of luminosity in summer and 24 h darkness in winter) and climatic conditions (e.g., low temperatures, storms, and white-out situations) is compounded by the difficulties of long displacements on rugged and snow-covered terrain and communication restrictions [13]. Additional hindrances facing the Antarctic ICE are punctual and occupation-specific and are also of interest to investigative physiology research; one example is the divers who submerge themselves in the icy waters for routine inspection and repair in ship hulls and whose cardiovascular and thermoregulatory adjustments are objects of study for exercise physiology.

Despite the increasing gain in technology and logistical expertise, the data collection in Antarctic expeditions is defiant. Prior planning and shipment of all necessary materials occur months before a research expedition. Moreover, due to the field conditions for data collection, especially in wild camping, several unexpected problems might appear regarding nature intemperate (e.g., storms lasting hours to days that prevent field trips and loss of tents due to strong winds). To these difficulties, one adds the expected technological restraints, such as Internet signal absence and electricity supply only for short periods during the day (a few hours) by a fuel generator. Thus, for use during field expeditions in Antarctica, a device should have the following essential characteristics: (i) portability, battery life, and a low need for recharging the device; (ii) the possibility of data acquisition and transfer offline; (iii) an operating range up to low temperatures; and (iv) waterproof performance, as it must withstand snowstorms or even summer rains in the Antarctic Peninsula area. In addition, the difficulty in dissipating sweat during long walks in the field results in an excessive sweat accumulation on clothes, allowing only the use of technological tools operating soaked in water. Devices impermeability still guarantees data collection in other conditions, as with divers during immersion. In this context, novel wrist-worn wearables provide an opportunity to examine cardiac responses in the natural world where combinations of environmental stresses co-exist. Herein, this viewpoint presents the methodological experience for cardiac autonomic monitoring in the Antarctic, the specific demands for smart wearables to physiological tracking in ICE, and our growing expertise on its application. Besides, due to the restrictions imposed by COVID-19, we point out the necessity for investment in remote monitoring at research stations in Antarctica, which cannot substitute in situ monitoring but will be increasingly demanded from now.

## 2. Methods and Challenges for Cardiac Monitoring in the Antarctic Field during Camps

We assess heart function and cardiovascular autonomic control by carrying out heart rate (HR) and heart rate variability (HRV) measures. The measurements take place through a punctual record under baseline conditions (i.e., with the volunteer in a standardized resting condition) [4] and a full day’s work long recording [14], or over 24 h. This protocol allows us to characterize the basal cardiac responses and evaluate effort intensity, particularly during fieldwork. Mainly, we are interested in monitoring over time (from two weeks to two months in camps, and throughout the year in a research station) by recording the same variables at known intervals, such as weekly, fortnightly, or monthly, to obtain repeated measurements (as performed in [4]).

During HR and HRV measures, it is necessary to consider the psychostimulant effects of caffeine [15,16], nicotine [17], and alcohol [18]. Usually, volunteers are instructed to abstain from ingesting these substances 24 or 48 h before baseline data collection. However, in longer-term measures, food and beverages, in general, are not restricted, which reduces the control of intervening factors but contributes to the ecological validity of the data. Nonetheless, in both cases, the measures upon waking up are suitable parameters to be observed, as they are under less effect of feeding. In addition, it is necessary to consider the effect of possible cardiac disorders on HRV analysis. In conditions of arrhythmias, atrioventricular block, and presence of an implantable pacemaker, the HRV analysis does not reflect the autonomic modulation of the heart [19,20], limiting the use of this analysis. Other operational problems include the displacement of the chest strap (especially when sleeping), interference in the HRV, and failures in the signal capture that are detectable during the visual inspection of the data. Given the possibility of these occurrences, in point-based measures, we acquire data for a longer time (in general, twice as long) than would be used for data analysis (as shown in Figure S1, https://figshare.com/s/5438e50e26965250b0e6 (accessed on 5 February 2021)). This additional time allows the disposal of non-physiological data.

In a typical data collection day, the experimental proceedings to HR and HRV basal recording are carried out between 6:00 and 9:00 and do not change the volunteers’ routine. HR and HRV are determined with the volunteers remaining comfortably seated on a chair in a sheltered environment—inside the stations, ships, and at camp. In the latter condition, a tent is previously heated, and we ask the volunteers to wear coats to ensure thermal comfort. The RR intervals are continuously recorded during the last 10 min of rest, and, usually, 5 min are analyzed. We give preference to use a chest strap with small electrode pads to collect ECG signals because optical sensors are dependent on adhesion to the skin and susceptible to light and motion artifacts, reducing their accuracy with increasing exercise load [21,22,23]. Usually, we register RR intervals by a Polar H10 chest strap ECG synchronized with: (i) Polar^®^ S810i (Polar, Finland); (ii) Polar V800 (model before Polar VantageV2, Polar, Finland); or (iii) via Bluetooth with a smartphone application (app), such as HRV Expert (CardioMood) or EliteHRV. Obtaining baseline measurements is relatively simple, as the registration time is short. From all the described devices, it is possible to obtain raw data in ASCII-files (e.g., .txt or .csv) for HRV analysis in software as Kubios (Kubios Oy, Kuobios, Finland) that has premium and free versions.

With the evolution of different equipment and sensors, many of them are integrated with online pathways to cloud resources and storage, resulting in limitations of offline resources, such as data transfer. For this reason, offline equipment, such as Polar^®^ S810i (Polar, Finland) with wireless data transfer independently of the Internet (infrared communication), has advantages over more modern sensors during data collection in the ICE field, even in point-oriented measures. The offline data transfer guarantees the visualization of the data still in the field and the backup of the data in different equipment (pen drives and computers); thus, it is a noticeable advantage over devices that in offline mode only store data. For the latter, the synchronization with the Internet occurs after the expedition returning, which can take up to 2–3 months. Besides, it is always worth mentioning the concern of keeping data on a device for synchronization after three months of data collection, as camping in Antarctic environmental extreme conditions increases the risk of equipment damage. Therefore, in the opposite way of the market that increasingly demands online-synchronized devices, for field research in remote locations, the possibility of accessing data by a USB pen-drive or Bluetooth connection regardless of Internet access is an advantage.

An extra challenge is the 24-h HR record in the field, a period to obtain information on the circadian rhythm, and the sleep changes compared to daytime activities. Long-term measures require greater autonomy of devices, which should record at least one entire night of sleep without the need to download the data (for devices with offline transfer). For some Internet-dependent data transfer watches, e.g., V800 or Polar VantageV2, 40-h data recording in internal memory is possible, enabling one full day recording per unit. However, considering that we carry out fieldwork with 7–10 volunteers, with repeated measures, this option is ineffective and expensive, as we would have to transport many watches to the field.

One solution for 24-h HR access is the Polar H10 chest strap associated with the Polar Beat app. By triggering the recording by Polar Beat, the HR is saved on the H10. This relatively cheap data recording method on the chest strap independently of a watch is safer in an extreme environment as the equipment remains protected by the participants’ clothes. Additionally, since we monitor activity patterns (actigraphy) and skin temperatures, a chest strap recorder leaves wrist and hand body surfaces for this monitoring. A disadvantage is that Polar Beat records only the last about 8–10 h each, generating three log files per day for each volunteer (Figure S2, https://figshare.com/s/5438e50e26965250b0e6 (accessed on 5 February 2021)), and each H10 needs one smartphone to synchronize data. Bearing in mind that device recharge is also a limitation in the Antarctica field because power energy depends on the generator, the need for several smartphones offers additional difficulty.

For 24-h HRV monitoring, a standard resource used by long-term clinical studies is ECG patch electrodes. Nevertheless, it is necessary to consider the usual problems for this measure, as the influence of movements by adding artifacts within the dataset [24]. Movements are frequent in a field situation in Antarctica, where the intensity of effort reaches 70–90% of the maximum HR during field displacements [14]. To identify and exclude moments with excessive movements, accelerometer data should be collected together with the HRV. It is worth noting that it is possible to perform other measurements in conjunction with ECG patch devices [25,26]. However, it is necessary to consider the electromagnetic effect on increased background noise upon the ECG signal [27,28]. To avoid impairment ECG interpretation, electromagnetic disturbance sources (e.g., a mobile phone) should not be close to the ECG, as in a breast pocket [28]. In addition, as already noted, the equipment must operate in extreme humidity. Furthermore, some ECGs rely on adhesives for their fixation, which may require hair removal and skin sanding, causing epidermal irritation and additional discomfort to the volunteers. Technological solutions are waterproof ECG options with additional monitoring features, e.g., Bittium Faros (Bittium Corporation, Oulu, Finland) and Actiheart (Cambridge Neurotechnology Ltd., Papworth, Cambridge, UK). The latter is even adaptable to chest straps. Notwithstanding these strengths for long-term ECG measures, it is necessary to consider these technologies’ relatively expensive cost, up to 15–40 times more than an H10 chest strap, depending on the ECG electrodes model. For some models, it is still required to evaluate reliability and validity in extreme climatic conditions in situations of effort in the field.

## 3. Specific Demands for a Smart Wearable to Physiological and Health Tracking in ICE-Environments

Looking ahead to a validated and waterproof built-in memory chest strap device for 24-h ECG RR recording, a complete wearable pocketknife [29] to obtain raw data in a single ASCII file offline (independent of the internet) would be an effective solution for fieldwork in Antarctica.

As researchers, we are interested in the facilitation of the comprehension of adaptive psychophysiological responses, including advances on the monitoring of HR and HRV for days or weeks, concomitantly with activity/sleep pattern recordings in synchrony with respiration rate, skin temperature, sweetness measurement (by galvanic skin response), blood pressure, volume pulse, and oxygen saturation (all by plethysmography), along with geolocation. Further enhanced health monitoring could be achieved by including an event button and an attached voice recorder. That would enable the participant to report by voice time-by-time occurrences (e.g., medication use, beginning and end of sleep, and performing physical exertion in the field) and perceptions (e.g., pain, thermal perception, sleepiness, and mood), with the event marking appearing in the records of all data. This unique multifunctional and compact all-in-one wearable physiological sensor might be a watch or armband; however, given the greater precision of the RR by ECG electrodes and the importance of core body temperature and sweat, chest straps or conjugated devices (armband plus chest strap) may be preferable solutions for scientific use.

Emerging development initiatives point to multifunctionality, as noted in the Multi-Sense CardioPatch [30,31], which must be tested during physical effort and under extreme climatic conditions.

## 4. Increased Remote Monitoring Demand Due to COVID-19 Restrictions

With the outbreak of COVID-19, Brazilian researchers decided not to move to Antarctica between October 2020 and March 2021. To minimize the impacts of social isolation in the research carried out in Antarctica and, for the sake of continuity of the health data collection, we maintained the remote monitoring in the overwinter Brazilian group responsible for logistics operation of the Antarctic Station Comandante Ferraz. In general, cardiac monitoring at research stations does not impose limitations because they have a stable power supply and Internet structure. Usually, for collecting cardiovascular autonomic data, the researchers distribute the chest straps and watches. After the registration period, the equipment synchronizes with the app for online data loading and remote analysis. These data transfer proceedings are simple for expert researchers, but it requires time or imposes complexity and an additional mental burden on the participants. In this sense, the pandemic revealed the need for monitoring that depends only on the volunteers’ use of the wrist-worn device, without having to do any operation for synchronization. Indeed, one demand is a programming device previously connected via the Internet to a data page that automatically updates daily data. Although previously programmed device demand was revealed only at this point, this implementation could contribute to telemedicine in ICE. In addition to obtaining data to understand adaptive responses, a time-by-time RR and ECGs wireless data transmission can be a diagnostic tool of critical arrhythmic events. In this way, even though thousands of kilometers away, the real-time results at the disposal of researchers and the health team would contribute to the wellness management of individuals who stay for a year in Antarctica.

## 5. Conclusions

Although Antarctica’s extreme conditions pique physiologists’ interest by influencing cardiac autonomic control, ICE conditions impose limitations on record and monitor biosignals. Data collections in ICE demand portable or wearable devices resistant to weather conditions and relatively independent of the technological facilities utilized in urbanized locations (e.g., internet and electricity).

Point-oriented HR and HRV measures are relatively simple to perform in the field using commercial devices. However, transiting from point-oriented to continuous measurements, the limitations imposed by the remote and extreme Antarctica are even more decisive for the choice and operation of a device, reducing wearable tools’ feasibility with comfort for the volunteers and validation use during physical effort and in extreme conditions. To move forward, the biosignal monitoring in extreme physiology requires technical advances in devices able to operate offline, as a wearable waterproof pocketknife with built-in memory for 24-h RR recording, followed by an endeavor for its validation under different conditions. In addition, the current COVID-19 pandemic imposed additional restrictions even on more accessible locations in Antarctica—the researchers’ stations—increasing the demand for pre-programmed devices, which can potentially contribute to telemedicine extend monitoring to predict changes over time.

In summary, the continued development of wearable technologies that operate in the face of ICE obstacles is essential for improving the quality of data obtained in the field—and this demand will continue increasing. Assuredly, wearables advances will increasingly contribute to the achievement of outcomes, allowing the development of interventions for the individuals’ well-being in ICE.

## Data Availability

Data is contained within the Supplementary Materials. The data presented in this study are available in [https://figshare.com/s/5438e50e26965250b0e6 (accessed on 5 February 2021)].

## Supplementary Materials

The following are available online at https://www.mdpi.com/1424-8220/21/4/1303/s1, Figure S1: Cardiac autonomic data of RR time series, Figure S2: Cardiac autonomic data of HR over 24-h.
