# Supplementary material for: Smart Wearables for Cardiac Autonomic Monitoring in Isolated, Confined and Extreme Environments: A Perspective from Field Research in Antarctica
Source: Sensors (Basel). 2021 Feb 11;21(4):1303. doi: 10.3390/s21041303 (PMC7917677; doi:10.3390/s21041303)
Supplement: Supplementary file 1 [file sensors-21-01303-s001.pdf]

Cardiac Autonomic Data Records Obtained during Field Data Collection in Antarctica

Michele M. Moraes, Thiago T. Mendes and Rosa M. E. Arantes

This is supplemental material for the viewpoint: Moraes, M.M., Mendes, T.T., Arantes, R.M.E. Smart wearables for cardiac autonomic monitoring in isolated, confined and extreme environments: A perspective from field research in Antarctica.

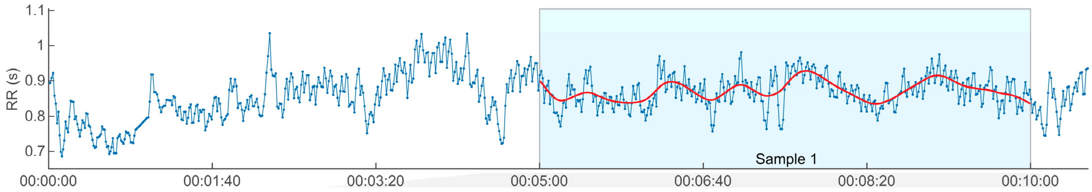

Figure 1. Cardiac autonomic data of RR time series. Representative (n=1) recording of RR time series measured during 10 minutes of rest (baseline) by chest strap H10 and Polar S810 (Polar, Finland). Selection highlighted in blue refers to the 5-minute interval used for the analysis of heart rate variability. Visualization in Kubios (Kubios Oy, Kubios, Finland)

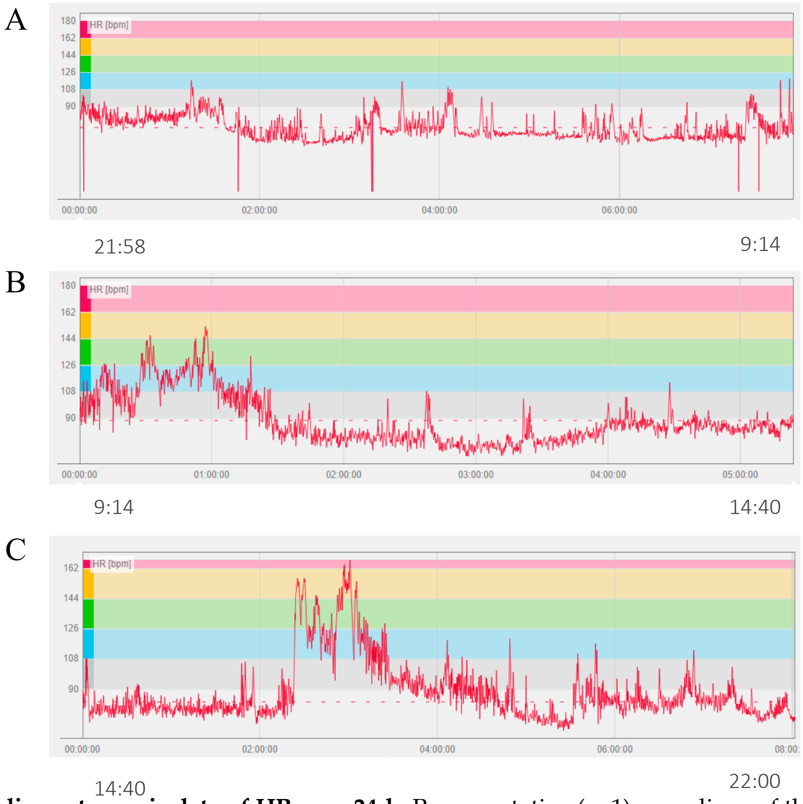

Figure 2. Cardiac autonomic data of HR over 24-h. Representative (n=1) recordings of three log files over 24-h heart rate (HR) measures through the chest strap H10 and the Polar Beat app (Polar, Finland), with data obtained: A) between 21:58 h of a first-day measure until 09:14 h of the following day, B) between 9:14 h and 14:10 h, C) between 14:40 h and 22:00 h. Visualization in Polar Flow app (Polar, Finland). The colors correspond to different ranges of HR values; gray: 90 to 108 bpm (49% to 59% of the maximum HR, HR_{MAX}, of the individual), blue: 108 to 126 bpm (59% to 68% HR_{MAX}); green: 126 to 144 bpm (68% to 78% HR_{MAX}); yellow: 144 to 162 bpm (78% to 88% HR_{MAX}) and red: 162 to 180 bpm (78% to 98% HR_{MAX}).
